# Suppressive Effect of Two Cucurbitane-Type Triterpenoids from *Momordica charantia* on *Cutibacterium acnes*-Induced Inflammatory Responses in Human THP-1 Monocytic Cell and Mouse Models

**DOI:** 10.3390/molecules26030579

**Published:** 2021-01-22

**Authors:** Lu-Te Chuang, Wen-Cheng Huang, Yu-Chen Hou, Jong-Ho Chyuan, Hsiang Chang, Chi-I Chang, Tsung-Hsien Tsai, Po-Jung Tsai

**Affiliations:** 1 Department of Biotechnology and Pharmaceutical Technology, Yuanpei University of Medical Technology, Hsinchu 300, Taiwan; ltchuang@mail.ypu.edu.tw (L.-T.C.); hchang@mail.ypu.edu.tw (H.C.); 2 Department of Human Development and Family Studies, National Taiwan Normal University, Taipei 106, Taiwan; wencheng7373@gmail.com; 3 Master Program in Food Safety, College of Nutrition, Taipei Medical University, Taipei 110, Taiwan; ychou@tmu.edu.tw; 4 Hualien District Agricultural Research and Extension Station, Hualien 973, Taiwan; jonghoc@hdares.gov.tw; 5 Department of Biological Science and Technology, National Pingtung University of Science and Technology, Pingtung 912, Taiwan; changchii@mail.npust.edu.tw; 6 Department of Dermatology, Taipei Municipal Wan Fang Hospital and Taipei Medical University, Taipei 116, Taiwan; 7 Program of Nutrition Science, School of Life Science, National Taiwan Normal University, Taipei 116, Taiwan

**Keywords:** acne, *Cutibacterium acnes*, *Momordica charantia*, anti-inflammation, kuguacin R, 3β,7β,25-trihydroxycucurbita-5,23-dien-19-al (TCD)

## Abstract

*Cutibacterium acnes* (formerly *Propionibacterium acnes*) is one of the major bacterial species responsible for acne vulgaris. Numerous bioactive compounds from *Momordica charantia* Linn. var. *abbreviata* Ser. have been isolated and examined for many years. In this study, we evaluated the suppressive effect of two cucurbitane-type triterpenoids, 5β,19-epoxycucurbita-6,23-dien-3β,19,25-triol (Kuguacin R; KR) and 3β,7β,25-trihydroxycucurbita-5,23-dien-19-al (TCD) on live *C. acnes*-stimulated in vitro and in vivo inflammatory responses. Using human THP-1 monocytes, KR or TCD suppressed *C. acnes*-induced production of interleukin (IL)-1β, IL-6 and IL-8 at least above 56% or 45%, as well as gene expression of these three pro-inflammatory cytokines. However, a significantly strong inhibitory effect on production and expression of tumor necrosis factor (TNF)-α was not observed. Both cucurbitanes inhibited *C. acne*s-induced activation of the myeloid differentiation primary response 88 (MyD88) (up to 62%) and mitogen-activated protein kinases (MAPK) (at least 36%). Furthermore, TCD suppressed the expression of pro-caspase-1 and cleaved caspase-1 (p10). In a separate study, KR or TCD decreased *C. acne*s-stimulated mouse ear edema by ear thickness (20% or 14%), and reduced IL-1β-expressing leukocytes and neutrophils in mouse ears. We demonstrated that KR and TCD are potential anti-inflammatory agents for modulating *C. acnes*-induced inflammation in vitro and in vivo.

## 1. Introduction

Acne vulgaris (acne) is a chronic inflammatory skin disease strongly associated with excessive sebum production, abnormal keratinization, and inflammatory responses to bacterial colonization [1]. It is well-accepted that residential bacteria of skin sebaceous glands, such as Gram-positive anaerobic *Cutibacterium acnes* (formerly *Propionibacterium acnes*) play the critical role in the development of acne [2]. *C. acnes* secretes hydrolytic enzymes such as proteases, lipases, and hyaluronidases to irritate and damage skin tissues, leading to cutaneous inflammation, infection, and immune responses. Recent studies indicated that pro-inflammatory cytokines secreted from keratinocytes, sebocytes, and monocytes, in response to *C. acnes* stimulation [3], attract immune cells to the infected pilosebaceous unit, and cause chronic inflammation [2,4]. Since excessive secretion of pro-inflammatory mediators has been highly related to acne pathogenesis, developing a strategy-based approach to suppress *C. acnes*-induced inflammatory responses might be applicable to alleviate this chronic inflammatory skin disease.

**Figure 1:**
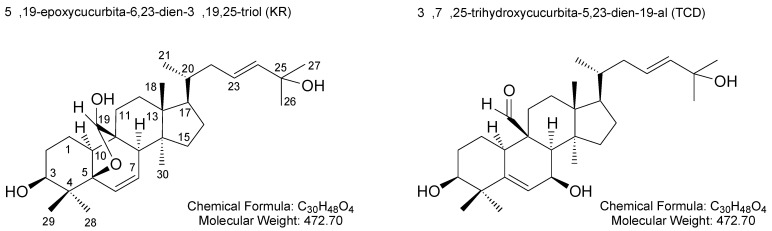
Chemical structures of 5β,19-epoxycucurbita-6,23-dien-3β,19,25-triol (Kuguacin R; KR) and 3β,7β,25-trihydroxycucurbita-5,23-dien-19-al (TCD).

*Momordica charantia* (bitter melon or bitter gourd) is a nutrient-rich vegetable cultivated in tropical and subtropical regions of the world. Cucurbitane triterpenoids, a group of unique tetracyclic triterpenoids characterized by a 19-(10 → 9β)-*abeo*-10α-lanost-5-ene ring skeleton, were originally obtained from the *Momordica* species [5]. Previously, 5β,19-epoxycucurbita-6,23-dien-3β,19,25-triol (Kuguacin R; KR) and 3β,7β,25-trihydroxycucurbita-5,23-dien-19-al (TCD) were isolated and collected from leaves of *M. charantia* L. var. *abbreviate* Ser. (wild bitter melon; WBM), a variety of bitter melon (Figure 1) [6].

These two cucurbitanes have been shown to suppress *Porphyromonas gingivalis*-induced inflammatory responses in the human THP-1 monocytic cell model, and could prevent periodontal disease progression in a mouse model of experimental periodontitis [6]. Furthermore, Bai and colleagues [7] reported that TCD significantly suppressed human MCF-7 breast cancer cell proliferation and induced cell apoptosis through the modulation of cell signaling cascades, such as protein kinase B (PKB or Akt)/nuclear factor-kappa B (NF-κB), p38 mitogen-activated protein kinases (MAPK), and p53 phosphorylation. However, due to the lack of commercially inexpensive availability of pure KR or TCD, limited studies have been conducted to reveal the mechanisms involved for the beneficial applications of these two triterpenoids.

To this end, we first evaluated the possible suppressive effect of KR and TCD on *C. acnes*-induced production and gene expression of pro-inflammatory mediators in human THP-1 monocytes. In addition, inflammatory signaling cascades were analyzed to determine the mechanisms for the action of both cucurbitane triterpenoids, including activation of myeloid differentiation primary response 88 (MyD88), MAPK, and caspase-1. Moreover, by using the *C. acnes*-inoculated mouse ear model, we examined the suppression effect of KR and TCD on *C. acnes*-induced inflammatory responses. The results of this study provided new aspects regarding in vitro and in vivo anti-inflammatory properties of KR and TCD.

## 2. Results and Discussion

### 2.1. Effects of KR and TCD on C. acnes-Induced Cellular Cytokine Production and mRNA Expression

**Figure 2:**
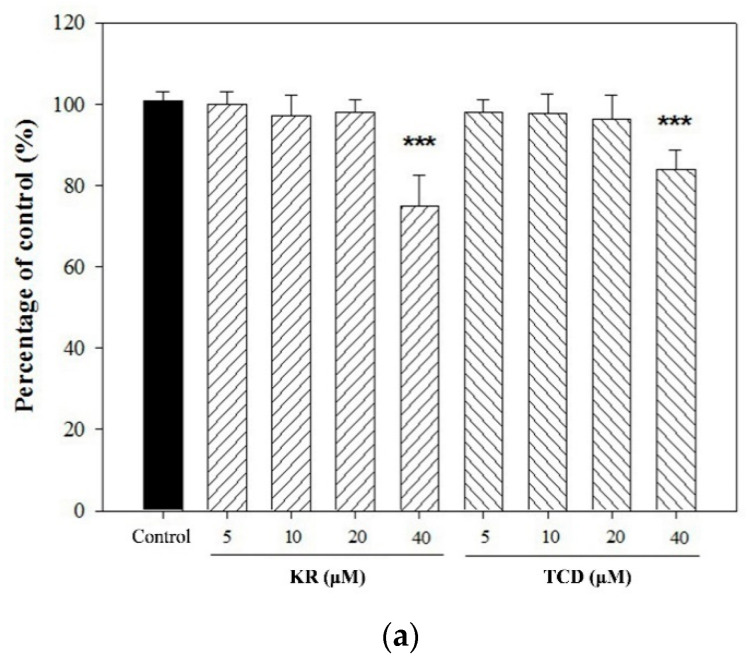

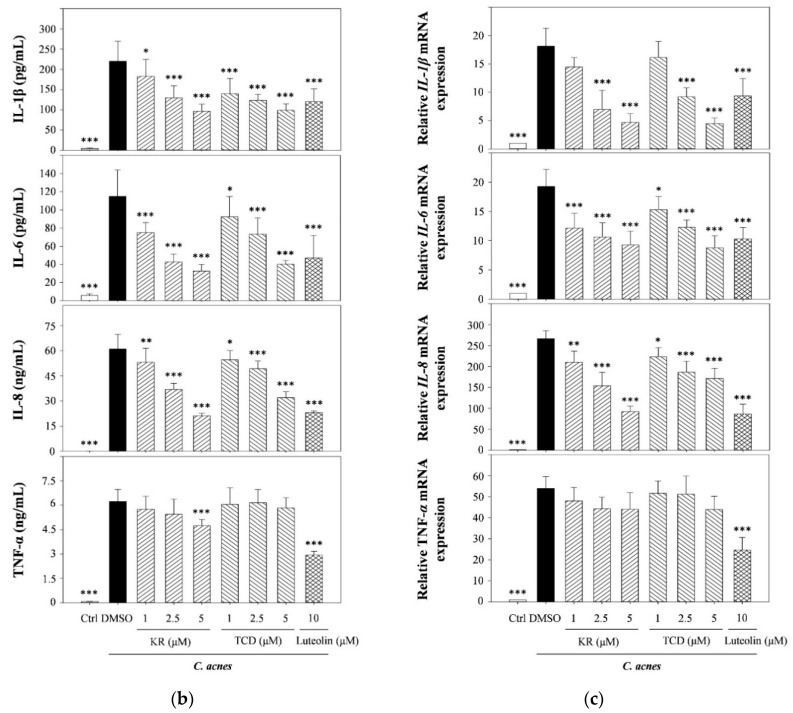
Effects of KR and TCD on cell viability of THP-1 cells (**a**) and *C. acnes*-induced production (**b**) and gene expression (**c**) of pro-inflammatory cytokine in human monocytic THP-1 cells. THP-1 cells were incubated with DMSO as the negative control, or co-cultured with *C. acnes* (M.O.I. = 75) and different concentrations (1, 2.5 or 5 μM) of KR or TCD for 24 h (production) (**a**) or 16 h (gene expression) (**b**). The cell-free culture supernatants were subsequently collected and analyzed for their content of IL-1β, IL-6, IL-8, and TNF-α. Each value represents the mean ± SD. Values with different symbols are significantly different from the *C. acnes* control (*C. acnes* alone) at *p* < 0.05 (*), *p* < 0.01 (**), and *p* < 0.001 (***).

To examine whether KR or TCD affected cell viability, THP-1 cells were incubated in culture medium supplemented with various concentrations (up to 40 μM) of tested samples. No adverse effect on cell proliferation was observed whenever the concentration of KR or TCD was below 20 μM (Figure 2a). To determine whether both cucurbitanes could suppress *C. acnes*-induced inflammatory responses, THP-1 monocytes were co-cultured with live *C. acnes* and in three levels (1, 2.5 or 5 μM) of KR or TCD. Figure 2b shows that the production of pro-inflammatory mediators (IL-8, IL-1β, IL-6 and TNF-α) was significantly elevated in response to *C. acnes* simulation. However, supplementation of the culture medium with different levels of KR significantly reduced respective cytokine production by as much as 56% (IL-1β), 71% (IL-6), 65% (IL-8), and 24% (TNF-α). Similarly, TCD significantly suppressed *C. acnes*-induced production of IL-1β (up to 60%), IL-6 (up to 45%), and IL-8 (up to 62%) (Figure 2b). In contrast, the production of TNF-α was not affected by TCD. Furthermore, we found that anti-inflammatory agent luteolin significantly inhibited *C. acnes*-stimulated induction of four cytokines in THP-1 cells. Luteolin, a flavone originally from numerous plants, has been determined to exert potent anti-oxidant and anti-inflammatory activities. For example, luteolin decreases adverse photobiological damages in the skin by acting as the first line of defense. Luteolin also exerts an anti-inflammatory effect on keratinocytes, fibroblasts, and immune cells by suppressing proinflammatory mediators and regulating signaling pathways. Recent evidence has demonstrated that luteolin could be applied with respect to inflammatory skin diseases, skin aging and skin cancer, wound healing, etc. [8,9]. Thus, luteolin was used as a positive control in this study.

We also determined that KR or TCD affected the expression of these same cytokines at the transcriptional level. Figure 2c shows that the expressions of pro-inflammatory IL-1β, IL-6 and IL-8 were significantly downregulated by KR and TCD. Only *C. acnes*-simulated mRNA levels of TNF-α were not reduced significantly by either cucurbitanes.

In this study, although we have reported that KR and TCD exert their anti-inflammatory properties by suppressing immune responses, we speculated that the decrease in pro-inflammatory cytokine production was also due to the anti-bacterial effect of both cucurbitanes. Based on results of the broth microdilution assay, the minimal inhibitory concentrations (MIC) of both cucurbitanes were greater than 100 μM; thus, the MIC was at least 20-fold higher than the highest concentration (5 μM) of both cucurbitanes, which we added into the THP-1 cell culture. Therefore, we confirmed that the anti-microbial potential of both cucurbitanes did not contribute to the decrease in *C. acnes*-induced pro-inflammatory mediator production. We previously demonstrated that KR and TCD inhibit heat-killed *P. gingivalis*-stimulated cytokine production by THP-1 cells [6]. In the present study, using the co-culture model of live *C. acnes* and THP-1 monocytes, we observed that both cucurbitanes suppress gene expression and production of pro-inflammatory mediators. These findings indicated that exposure of monocytes to live *C. acnes* or heat-inactivated *P. gingivalis* stimulated cellular inflammatory responses, and also that KR and TCD strongly suppressed bacteria-induced inflammation. In addition, since KR and TCD inhibited inflammatory responses triggered by Gram positive (*C. acnes*) and Gram negative (*P. gingivalis*) bacteria. We believe that these two cucurbitanes are potential candidates for developing novel therapeutic agents against bacteria-stimulated inflammation.

### 2.2. Effects of KR and TCD on Activation of MyD88, MAPK, and Caspase-1

**Figure 3:**
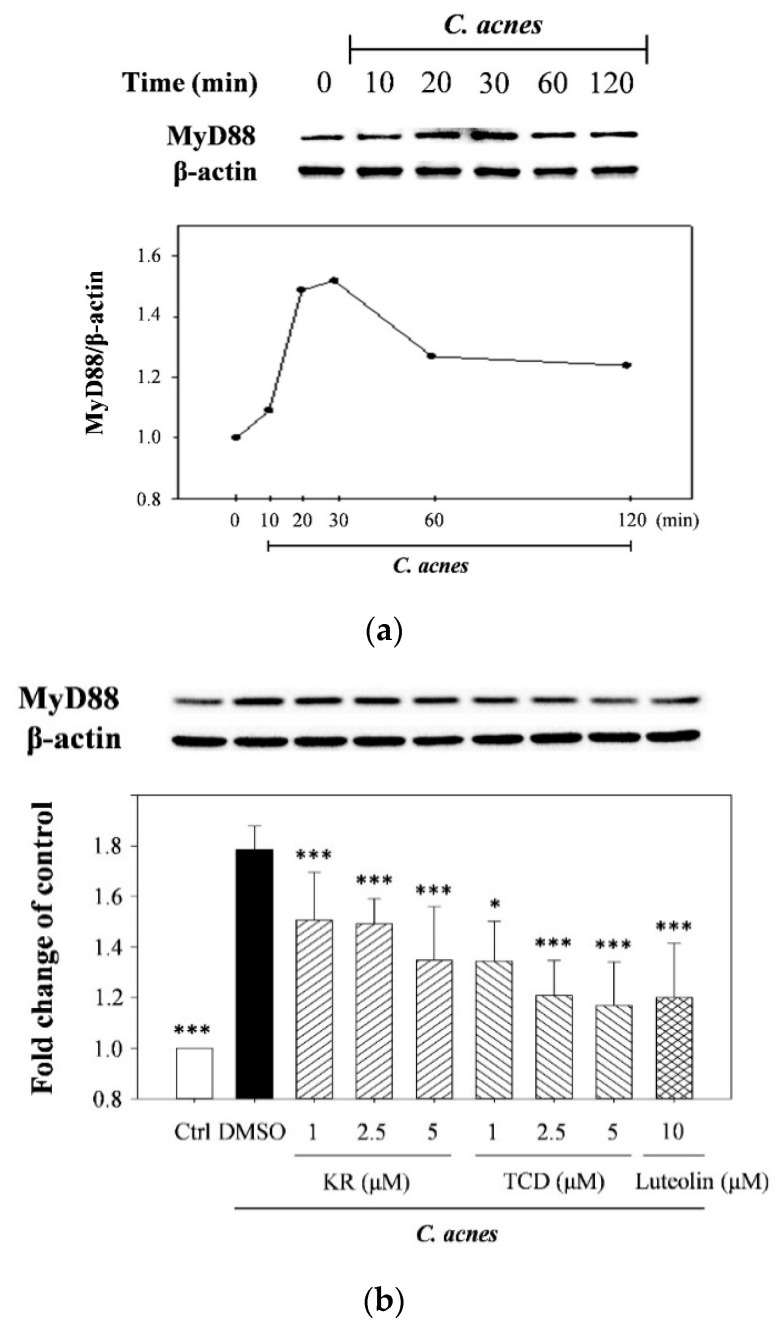

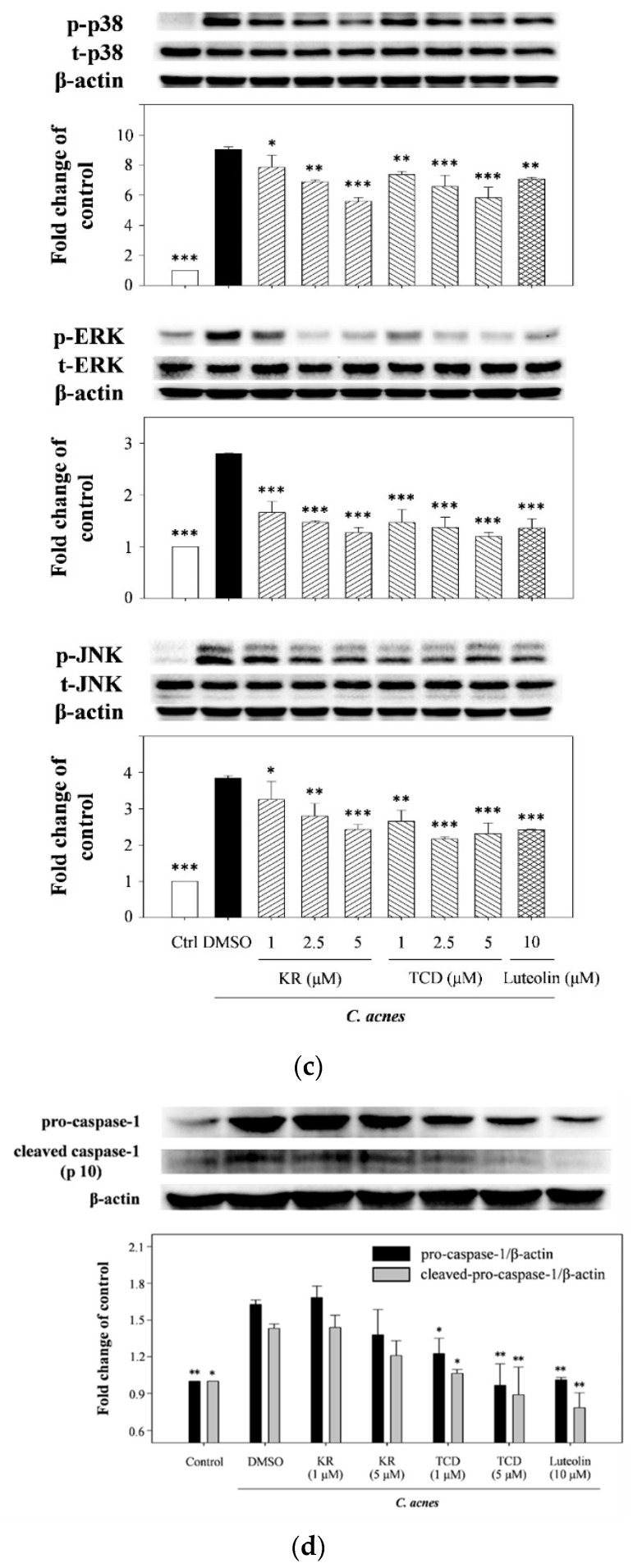
Effect of stimulation periods of *C. acnes* on activation of myeloid differentiation primary response 88 (MyD88) (**a**), and the effects of KR or TCD on MyD88 (**b**), mitogen-activated protein kinases (MAPK) (**c**), and on caspase-1 (**d**) expression. THP-1 cells were cultured with DMSO and co-incubated with *C. acnes* (M.O.I. = 75) for various periods of time (0, 10, 20, 30, 60, and 120 min) (**a**). To determine activation of MyD88, MAPK, and caspase-1, cells were cultured with DMSO as the negative control, or co-incubated with *C. acnes* (M.O.I. = 75) and different concentrations (1, 2.5 or 5 μM) of KR or TCD for 20 min (for MyD88) (**b**), 2 h (for MAPK) (**c**), or 16 h (for caspase-1 expression activation) (**d**). Each value shows that the mean ± SD. Values with different symbols are significantly different from the *C. acnes* control (*C. acnes* alone) at *p* < 0.05 (*), *p* < 0.01 (**), and *p* < 0.001 (***).

*C. acnes* had been reported to stimulate production and gene expression of pro-inflammatory mediators by activating toll-like receptor-2 (TLR2)/MyD88-mediated MAPK signaling [10,11]. We therefore investigated whether KR or TCD might inhibit cytokine synthesis in monocytes by means of suppressing signal transduction cascades. Figure 3a shows the time-course of MyD88 activation in THP-1 cells in response to exposure to live culture of *C. acnes* for different stimulation times (0, 10, 20, 30, 60, and 120 min). Based on the optimal results, 20-min incubation was determined to stimulate cells for MyD88 determination. The results in Figure 3b show that the *C. acnes*-induced stimulation significantly raised the level of MyD88 (1.78-fold). However, activation of MyD88 in *C. acnes*-stimulated monocytes co-incubated with KR or TCD was significantly reduced to 75% or 62%. Luteolin suppressed the level of MyD88 activation by 33%. As for MAPK, the levels of phosphorylated p38-, ERK-, and JNK-MAPK in THP-1 cells were increased in response to stimulation of *C. acnes* (Figure 3c). KR or TCD suppressed the expression of all three phosphorylated MAPK proteins by 36–39% (p38), 55–58% (ERK), and 37–40% (JNK).

The possible role for IL-1β in the suppressive effect of both cucurbitanes on *C. acnes*-induced cytokine expression was then investigated. Caspase-1, a specific cytosolic protease also known as interleukin-1β converting enzyme (ICE), is the rate-limiting enzyme in the production of IL-1β from its precursor, pro-IL-1β, by means of the proteolytic cleavage of a 14-kDa peptide [12]. Figure 3d shows that pro-caspase-1 (inactive zymogen form) and the caspase-1 p10 subunit (active form) were over-expressed in response to *C. acnes* stimulation. TCD significantly suppressed expression of pro-caspase-1 and cleaved caspase-1 (p10) by 41% and 32%, respectively, as compared to the *C. acnes*-treated control, whereas, KR lowered it only slightly at a concentration of 5 μM; however, these effects were not statistically significant. The discrepant effect on caspase-1 expression by both cucurbitanes could be due to their chemical structures. Comparing their structures, KR with a 5β,19-epoxy moiety in the skeleton, the three-dimensional structure of KR can form a steric bridge. In addition, the inhibitory activity of TCD with a carbonyl group at C-19 was stronger than that of KR with a hemiactal group at C-19. The carbonyl group at C-19 might play an important role in the modulatory effect on caspase-1 expression. However, further investigation is required to explore the relationship between their chemical structure and anti-inflammatory properties.

To date, the precise pathological process underlying *C. acnes*-mediated skin inflammation remains unclear. A series of bacterial and biochemical stimuli initiates a complex interaction between immune microenvironments and abnormal skin cell proliferation/function. For example, *C. acnes* produces numerous enzymes and cytotoxic chemicals that stimulate the innate immune responses through cellular pattern recognition receptors, such as toll-like receptors and nucleotide oligomerization domain (NOD)-like receptors to trigger the MyD88-mediated MAPK/NF-κB signaling cascades [11,13]. MyD88, an adaptor protein for IL-1 receptor (IL-1R)/TLR signaling, is widely recognized as the key component of many inflammatory pathways [14]. After membrane-bound receptors bind to their specific ligands, MyD88 spontaneously activates downstream MAPK and NF-κB cellular signaling, which is responsible for the over-expression and production of many inflammatory mediators, subsequently leading to an inappropriate and persistent immune activation and inflammatory responses [14]. In this study, we confirmed that both cucurbitanes significantly suppressed activation of both MyD88 and MAPK (Figure 3a–c), as well as the gene expression and production of four cytokines (Figure 2). These findings are in accordance with results of our previous reports that KR and TCD suppressed expression of phosphorylated MAPK in *P. gingivalis*-stimulated THP-1 cells [6]. These results also lead us to speculate that the potent anti-inflammatory properties of KR and TCD might be strongly associated with the molecular mechanisms underlying suppressive effects of the crude extracts or total phenolic extract (TPE) of WBM leaf on *C. acnes*-induced inflammatory responses [15,16].

### 2.3. Effects of KR and TCD on C. acnes-Stimulated Mouse Ear Edema

**Figure 4:**
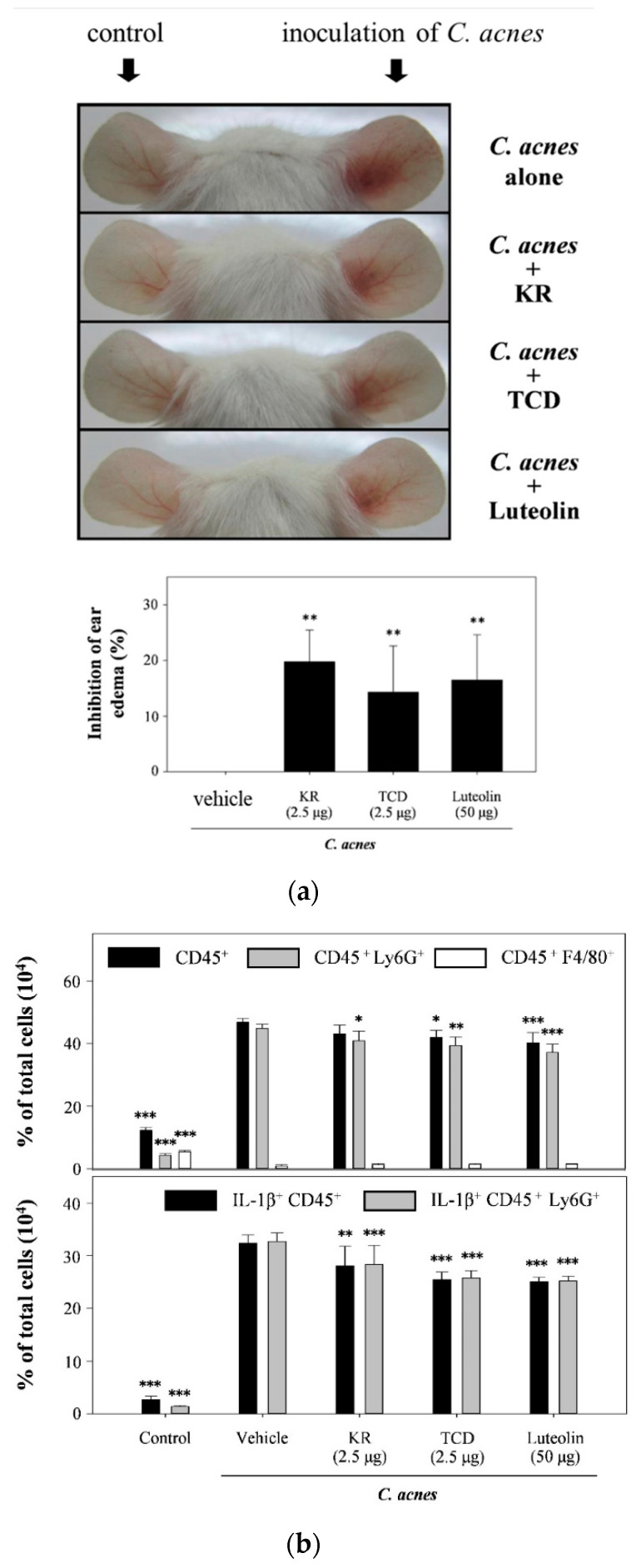

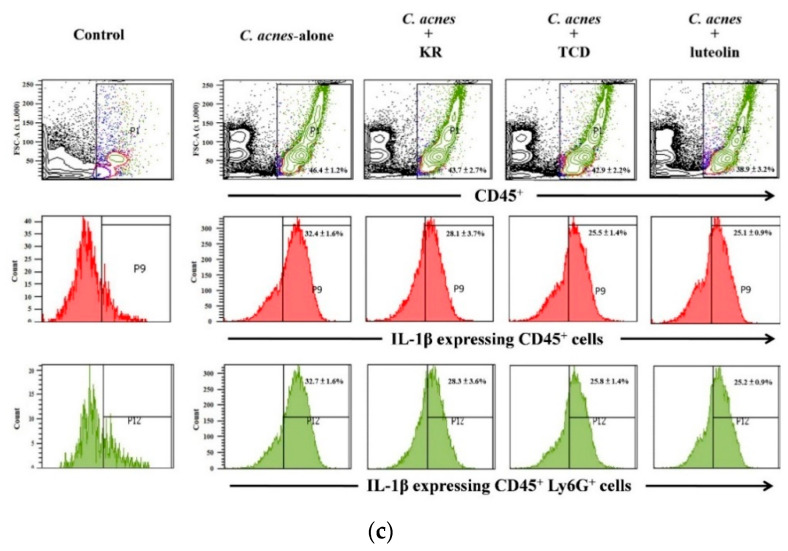
Effects of KR or TCD on *C. acnes*-induced mouse ear edema. PBS, KR (2.5 μg/site), TCD (2.5 μg/site), or luteolin (50 μg/site) were intradermally injected, followed by immediate injection of *C. acnes*. Twelve hours after the injection, the inhibitory effects of KR or TCD on *C. acnes*-induced ear swelling were assessed by measuring the ear thickness (**a**), and *C. acnes*-induced inflammatory cells in mouse ear was performed by flow cytometry analysis (**b**,**c**). Each value represents the mean ± SD. Values with different symbols are considered to be significantly different from the *C. acnes* control (*C. acnes* alone) at *p* < 0.05 (*), *p* < 0.01 (**) and *p* < 0.001 (***).

Our previous studies showed that injection of live *C. acnes* (6 × 10^7^ CFU/site) into mouse ears caused epidermal microabscesses and swelling as compared to the untreated group [16,17]. In this study, we inquired if KR and TCD affected inflammatory responses in the *C. acnes*-stimulated mouse ear edema model. In this study, we inquired if KR and TCD affected inflammatory responses in the *C. acnes*-stimulated mouse ear edema model. *C. acnes*-induced ear edema was attenuated by respective treatment of KR (20%), TCD (14%), or luteolin (17%) (Figure 4a). The results of flow cytometric analysis demonstrated that the proportion of leukocytes (CD45^+^) infiltrating into mouse ear tissue was significantly raised from 12% to 47% after 12-h of *C. acnes* inoculation (Figure 4b,c). Similarly, the proportion of neutrophils (CD45^+^Ly6G^+^), accounting for 96% of total inflammatory leukocytes, was markedly increased from 4% to 45%. Our observation is in accordance with the fact that neutrophils are the first and major subpopulation of leukocytes to respond at the site of injection in the early stage of inflammation [18]. Most macrophages derived from monocytes are attracted into traumatized tissue at a later stage of inflammation (the period of 24 to 72 h). Thus, only a small proportion of the infiltrating macrophages (CD45^+^F4/80^+^) in response to 12 h-stimulation was observed (Figure 4b,c). Co-injection of KR, TCD, or luteolin along with *C. acnes* stimulation reduced the proportions of infiltrated immune cells from 47% to 41% (leukocytes) or 45% to 37% (neutrophils), respectively. Furthermore, levels of both IL-1β-expressing leukocytes and neutrophils were also lowered from 33% down to 25–29%. Treatments of KR, TCD, or luteolin had no effect on the macrophage population, which could be explained by low infiltration of macrophages at 12-h of *C. acnes* inoculation.

Live *C. acnes* bacteria enhanced IL-1β production in human THP-1 cells through the activation of caspase-1 (Figure 3d). Our results reinforce previous findings showing that the upregulation of caspase-1 by *C. acnes* triggers the cleavage of pro-IL-1β, resulting in production and secretion of mature IL-1β from the cells [19,20]. In the investigation of mechanisms underlying inflammatory responses elicited by *C. acnes*, Qin and colleagues [21] found that *C. acnes* stimulates IL-1β production through the activation of NOD-like receptor protein 3 (NLRP3)-inflammasome and caspase-1 in human monocytes. They also detected increased amounts of caspase-1 and NLRP-3 in the dermis around pilosebaceous follicles in acne lesions. Rodriguez and colleagues [22] demonstrated that production of IL-1β is also well regulated by a TLR2-MyD88-NLRP3 signaling pathway in pathogen-infected murine bone marrow-derived macrophage and mouse models. In the present study, we documented that both cucurbitanes, KR and TCD, suppressed activation of MyD88 and expression of caspase-1 and IL-1β. In addition to inactivation of MAPK signaling, we speculate that KR and TCD might downregulate TLR2-mediated MyD88 signaling and NLRP3 inflammasome activation, leading to the inhibition of active caspase-1, subsequently to the reduction of IL-1β expression and production.

In this study, we have demonstrated that KR and TCD are as effective as anti-inflammatory luteolin in suppressing *C. acnes*-induced inflammatory responses. The molecular mechanisms underlying the suppression of *C. acnes*-induced inflammatory responses could be through the inhibition of TLR2-MyD88-mediated MAPK and NLRP3 inflammasome activation. Therefore, our findings raise the prospect that both KR and TCD have therapeutic potential as anti-inflammatory agents to alleviate or relieve inflammatory skin diseases. However, limited literature of the potentially beneficial applications of both unusual triterpenoids is published due to the shortage of inexpensive, pure KR and TCD compounds. After our methods for isolating and purifying both of these cucurbitanes [6] are available, future investigations on the toxicological and pharmaceutical evaluation of KR or TCD can be expected.

In conclusion, we have demonstrated that two cucurbitanes, KR and TCD suppress *C. acnes*-stimulated MyD88-mediated MAPK activation, thereby reducing the levels of pro-inflammatory mediators in THP-1 cells. Furthermore, two cucurbitanes relieved epidermal edema, immune cell infiltration, and IL-1β-expressing neutrophils in the mouse ear. Collectively, our findings indicated that KR and TCD exerted distinct anti-inflammatory effects, and that these two cucurbitanes can be considered as anti-inflammatory therapeutic candidates for moderating in vitro and in vivo inflammatory responses.

## 3. Materials and Methods

### 3.1. Materials

3-(4,5-dimethylthiazol-2-yl)-2,5-diphenyltetrazolium bromide (MTT), dimethylsulfoxide (DMSO), luteolin, and sodium dodecyl sulfate (SDS) were obtained from Sigma Chemical Co. (St. Louis, MO, USA). Fetal bovine serum (FBS), phosphate-buffered saline (PBS), RPMI 1640 medium, penicillin, and streptomycin were from Gibco (Carlsbad, CA, USA). Brain heart infusion (BHI) broth was purchased from Difco (Detroit, MI, USA). ELISA kits for detecting human interleukin (IL)-1β, IL-6, IL-8, and tumor necrosis factor-α (TNF-α) were supplied from Invitrogen (Carlsbad, CA, USA). All reagent-grade organic solvents were purchased from Burdick and Jackson (Muskegon, MI, USA). The antibodies used for Western blotting assay and flow cytometry were listed as Table 1.

### 3.2. Preparation of KR and TCD

Fresh WBM leaves (Hualien No.1) were obtained from the Hualien District Agricultural Research and Extension Station (Hualien, Taiwan). The leaves were washed, air-dried, and then ground to a fine powder. Using a series of extraction and partition techniques described elsewhere, KR and TCD were isolated, collected, and identified from WBM leaf extracts [6].

### 3.3. Bacterial and Cell Cultures

*C. acnes* strain BCRC10723 was obtained from the Bioresource Collection and Research Center (Hsinchu, Taiwan). *C. acnes* was cultured in BHI broth with 1% (*w/v*) glucose under anaerobic conditions in the BBL GasPak system (Becton Dickinson, Cockeysville, MD, USA). A human monocytic (THP-1) cell line BCRC60430 was also obtained from the Bioresource Collection and Research Center. Cells were maintained in RPMI1640 medium supplemented with 10% (*v/v*) heat-inactivated FBS, penicillin (100 U/mL), and streptomycin (100 µg/mL) at 37 °C in a humidified atmosphere with 5% CO_2_. To monitor cell viability of all treatments, the MTT method was applied.

### 3.4. Measurement of Cytokine Production in THP-1 Cells

A well-established co-culture model of *C. acnes* and THP-1 cells was used to investigate the anti-inflammatory properties of various herbal extracts on *C. acnes*-stimulated cytokine production [15,23]. The culture of *C. acnes* under log-phase was centrifuged (10,000× *g*; 5 min) and the cells were collected, washed thrice with PBS, and re-suspended in RPMI medium. THP-1 cells (1 × 10^6^ cells/mL per well) were seeded into 24-well plates with serum-free RPMI medium, and then co-cultured with live *C. acnes* (multiplicity of infection (M.O.I.) = 75) alone or in combination with KR, TCD or luteolin at 37 °C in a 5% CO_2_ humidified atmosphere. After incubation for 24 h, the cell-free supernatants were collected, and the levels of cytokines (IL-1β, IL-6, IL-8, and TNF-α) were determined using the corresponding enzyme immunoassay kits.

### 3.5. Analysis of mRNA Levels by Quantitative Real-Time Reverse Transcription Polymerase Chain Reaction (PCR)

Total RNA of human THP-1 cell samples was extracted and isolated using TRIzol reagent (Invitrogen; Carlsbad, CA, USA). The cDNA was then synthesized from the RNA with a reaction mixture of oligo (dT) primers and reverse transcriptase (Promega, Madison, WI, USA), following the manufacturer’s instructions. Real-time PCR analysis was conducted using an iCycler iQ Real-Time detection system (Bio-Rad, Hercules, CA, USA). To amplify cDNA, primer sets for IL-1β (forward primer, 5′-AAGCTGAGGAAGATGCTG-3′; reverse primer, 5′-ATCTACACTCTCCAGCTG-3), IL-6 (forward primer, 5′-GGAGACTTGCCTGGTGAAA-3′; reverse primer, 5′-CTGGCTTGTTCCTCACTACTC-3′), IL-8 (forward primer, 5′-TGCCAAGGAGTGCTAAAG-3′; reverse primer, 5′-CTCCACAACCCTCTGCAC-3′), TNF-α (forward primer, 5′-TCTTCTGCCTGCACTTTGG-3′; reverse primer, 5′-ATCTCTCAGCTCCACGCCATTG-3′) and glyceraldehydes 3-phosphate dehydrogenase (GAPDH) (forward primer, 5′-GTGAAGGTCGGAGTCAACG-3′; reverse primer, 5′-TGAGGTCAATGAAGGGGTC-3′) were used for PCR. To amplify respective cDNA, primer sets for target genes (IL-1β, IL-6, IL-8, TNF-α and glyceraldehyde-3-phosphate dehydrogenase (GAPDH)) and thermal cycling conditions applied for all PCR assays as described elsewhere [15]. The relative amounts of the PCR products were analyzed using iQ™5 optical system software (ver. 2.1; Bio-Rad). The mRNA contents of all targeted genes from all tested samples that were measured and analyzed after being normalized to those mRNA levels of GAPDH.

### 3.6. Western Blot Analysis

Human THP-1 cells were harvested, and total cellular protein was extracted from each sample, separated by 10% (*w*/*v*) sodium dodecyl sulfate polyacrylamide gel electrophoresis (SDS-PAGE), and then transferred onto a polyvinylidene difluoride (PVDF) (Millipore, MA, USA). Blots were processed using a primary antibody (1:1000 dilution of anti-human MyD88, MAPK, phospho-MAPK, pro-caspase-1 or cleaved caspase-1 or β-actin, followed by reaction with a secondary antibody (1:5000 dilution of immunoglobulin conjugated with horseradish peroxidase (Sigma). The immuno-reactive proteins of interest were analyzed using the enhanced ECL chemiluminescence Western blotting detection system (ChemiDoc XRS, Bio-Rad). Signal strengths were measured and quantified using densitometric program (Image Lab, Bio-Rad).

### 3.7. C. acnes-Induced Inflammation in Mouse Ears

We evaluated the protective effects of KR and TCD on *C. acnes*-stimulated inflammation in mouse ear edema model by using the method described elsewhere [13]. Male ICR mice (8 weeks old) were obtained from the Animal Center of the College of Medicine, National Taiwan University (Taipei, Taiwan). Mice were housed in a controlled environment and provided with a commercial chow diet (LabDiet, 5001, Purina, St Louis, MO, USA) and water ad libitum throughout the experiment. All procedures were performed in accordance with the Guide for the Care and Use of Laboratory Animals. The protocol was approved by the Institutional Animal Care and Use Committee (IACUC) of National Taiwan Normal University (IACUC Permit No. 104024). Mice were randomly divided into five groups (*n* = 5 per group): the normal control (control), inoculation of *C. acnes* (*C. acnes*-alone), inoculation of *C. acnes*/co-injection of TCD (*C. acnes* + TCD), inoculation of *C. acnes*/co-injection of KR (*C. acnes* + KR), and inoculation of *C. acnes*/co-injection of luteolin (*C. acnes* + luteolin). Live *C. acnes* (6 × 10^7^ CFU/10 μL in PBS) was intradermally injected into the right ear, while the left ears received an equal volume of PBS. Ten microliters of KR, TCD, or luteolin in PBS with 5% DMSO were injected into the identical site of both mouse ears immediately after *C. acnes* or PBS injection. Luteolin served as the control. Preliminary studies demonstrated that the dose of KR (2.5 μg/10 μL/site), TCD (2.5 μg/10 μL/site), or luteolin (50 μg/10 μL/site) was optimal for topical administration without causing obvious skin irritation (data not shown). As seen in the left ears of mice, which received TCD (2.5 μg/site), KR (2.5 μg/site) or luteolin (50 μg/site) in the absence of *C. acnes*, did not exhibit any apparent skin irritation after these treatments (Figure 4a).

Twelve hours after the injection, ear thickness was measured and recorded using a micro-caliper (Mitutoyo, Kanagawa, Japan). The extent of edema was evaluated based on the difference in thickness between both ears. The increase in ear thickness of the *C. acnes*-stimulated ear was expressed as a percentage of the PBS control. Mice were then sacrificed by carbon dioxide asphyxiation. Single-cell suspensions from *C. acnes*-induced skin lesions in mouse ears were prepared by a method described elsewhere [13]. Briefly, the ears were excised, divided into dorsal and ventral halves, and then dispersed into the RPMI medium. Single-cell suspensions were stained for 30 min with three fluorescently-conjugated antibodies, including PerCP-conjugated anti-mouse CD45 (a leukocyte marker), FITC-conjugated anti-mouse Ly6G (a neutrophil marker), and PE-conjugated anti-mouse F4/80 (a macrophage marker). For analysis of intracellular IL-1β, cells were first stained with PerCP-conjugated anti-mouse CD45 and FITC-conjugated anti-mouse Ly6G antibodies. Stained cells were then fixed, permeated with permeabilization buffer (eBioscience, San Diego, CA, USA), and incubated with an APC-conjugated anti-IL-1β monoclonal antibody, according to the manufacturer’s instructions. After incubation with probes, cells were suspended in staining buffer (PBS with 0.5% bovine serum albumin) and analyzed with a FACS Canto II flow cytometer (BD Biosciences, San Jose, CA, USA).

### 3.8. Statistical Analysis

Statistical data analyses were performed using the SPSS Statistics for Windows, version 19.0 (SPSS Inc., Chicago, IL, USA). Statistical significance was assessed using one-way ANOVA followed by LSD multiple range test. All data are collected and presented as means ± SD. Mean differences among groups were considered as statistically significant at the *p* ≤ 0.05 levels.

## Figures and Tables

**Table 1:** Antibodies used for Western blotting (WB) and flow cytometry (Flow).

Protein	Clone	Source/Company (Catalog Number)	Assay
MyD88	D80F5	Cell Signaling Technology (4283)	WB
p38	Polyclonal	Cell Signaling Technology (9212)	WB
ERK	137F5	Cell Signaling Technology (4695)	WB
JNK	56G8	Cell Signaling Technology (9258)	WB
p-p38	D3F9	Cell Signaling Technology (4511)	WB
p-ERK	20G11	Cell Signaling Technology (4376)	WB
p-JNK	81E11	Cell Signaling Technology (4668)	WB
pro-caspase-1	EPR4321	GeneTex (GTX62815)	WB
cleaved caspase-1	Polyclonal	GeneTex (GTX134551)	WB
β-actin	AC-74	Sigma (A5316)	WB
PerCP-anti-mouse CD45 ^1^	30-F11	BioLegend (103129)	Flow
FITC-anti-mouse Ly6G ^2^	1A8	BioLegend (127605)	Flow
PE-anti-mouse F4/80 ^3^	30-F11	BioLegend (127605)	Flow
APC-anti-mouse-IL-1β ^4^	NJTEN3	BioLegend (127605)	Flow

^1^ Peridinin chlorophyll protein (PerCP)-conjugated anti-mouse CD45. ^2^ Fluorescein isothiocyanate (FITC)-conjugated anti-mouse Ly6G. ^3^ Phycoerythrin (PE)-conjugated anti-mouse F4/80. ^4^ Allophycocyanin (APC)-conjugated anti-IL-1β.

## Data Availability

Not available.

## Sample Availability

Samples of the compounds are unavailable from the authors.
